# MRI and CBCT image registration of temporomandibular joint: a systematic review

**DOI:** 10.1186/s40463-016-0144-4

**Published:** 2016-05-10

**Authors:** Mohammed A. Q. Al-Saleh, Noura A. Alsufyani, Humam Saltaji, Jacob L. Jaremko, Paul W. Major

**Affiliations:** Department of Dentistry, Faculty of Medicine and Dentistry, University of Alberta, Edmonton, Canada; Department of Oral Medicine and Diagnostic Sciences, College of Dentistry, King Saud University, Riyadh, Saudi Arabia; Department of Radiology and Diagnostic Imaging, Faculty of Medicine and Dentistry, University of Alberta, Edmonton, Canada

**Keywords:** Multimodality, Registration, MRI, CBCT, CT, TMJ, TMJ disc

## Abstract

**Purpose:**

The purpose of the present review is to systematically and critically analyze the available literature regarding the importance, applicability, and practicality of (MRI), computerized tomography (CT) or cone-beam CT (CBCT) image registration for TMJ anatomy and assessment.

**Data sources:**

A systematic search of 4 databases; *MEDLINE, EMBASE, EBM reviews and Scopus*, was conducted by 2 reviewers. An additional manual search of the bibliography was performed.

**Inclusion criteria:**

All articles discussing the magnetic resonance imaging MRI and CT or CBCT image registration for temporomandibular joint (TMJ) visualization or assessment were included.

**Results and included articles’ characteristics:**

Only 3 articles satisfied the inclusion criteria. All included articles were published within the last 7 years. Two articles described MRI to CT multimodality image registration as a complementary tool to visualize TMJ. Both articles used images of one patient only to introduce the complementary concept of MRI-CT fused image. One article assessed the reliability of using MRI-CBCT registration to evaluate the TMJ disc position and osseous pathology for 10 temporomandibular disorder (TMD) patients.

**Conclusion:**

There are very limited studies of MRI-CT/CBCT registration to reach a conclusion regarding its accuracy or clinical use in the temporomandibular joints.

**Electronic supplementary material:**

The online version of this article (doi:10.1186/s40463-016-0144-4) contains supplementary material, which is available to authorized users.

## Background

Merging different imaging modalities such as magnetic resonance imaging (MRI), multi-detector computed tomography (CT) and Positron emission tomography (PET) to display both osseous and soft tissues has been undertaken for about 20 years in neurosurgery [1]. Digital registration tools were employed to optimize image alignment. Other medical applications of image registration have been introduced including computer-aided robotic orthopedic surgeries and radiotherapies [2–4].

Image superimposition to evaluate changes in facial soft tissues, skeleton and dentition has been performed for many years using two-dimensional (2D) radiographs [5, 6]. However, the 2D radiographs suffered many limitations such as tissue overlapping, landmark obstruction, distortion, magnification and object displacement. The contribution of three-dimensional (3D) cone-beam CT (CBCT) to the field of dentistry is significant especially for diagnosis, treatment planning of craniofacial structures and assessment of the hard tissues of the temporomandibular joint (TMJ) [7, 8]. CBCT overcame the limitations of 2D radiography and allows 3D image superimposition. CBCT superimposition using anatomical landmarks in the skull base to analyze changes in craniofacial bones and airway tract has been validated [9–11]. Virtual 3D surface models have been developed to quantify tissue displacement between two time points using a color-coded scale [12, 13]. Registration of CBCT images has evolved into automatic superimposition of 2 CBCT images using the mutual information registration concept and has recently been introduced as a new tool to evaluate the craniofacial changes and TMJ assessment [14, 15].

In 1998, Nebbe et al. superimposed sagittal MRI to lateral cephalometric radiographs to evaluate the temporomandibular joint (TMJ) disc position [16]. CBCT and MRI are the most commonly used diagnostic imaging techniques used in the field of dentistry. CBCT is optimum for viewing skeletal and dental tissues, and MRI is the standard for viewing masticatory muscles, ligaments and the cartilagenous disc of TMJ. Unlike registration of serial CBCT images, multimodality image registration between MRI and CBCT is challenging due to differences in voxel size, pixel intensity, anatomical structure identification, image orientation and field of view (FOV). Nevertheless, this registration is desirable as it provides a complementary image of soft and hard tissues in one picture frame for optimum diagnosis, treatment planning, and evaluation of treatment outcome.

The purpose of the present review is to systematically and critically analyze the available literature regarding importance, applicability, and practicality of MRI, CT and CBCT image registration for TMJ anatomy and assessment.

## Materials and methods

### Search strategy

Systematic search of four major databases, MEDLINE (1946 to 2015 Jan 10), All EBM Reviews-Cochrane DSR, DARE, and American College of Physicians Journal Club (1980 through January 13, 2016), Scopus (1965 through Jan 18, 2016), and EMBASE (1974 to 2016 January 18), [3] was conducted without language limitation. The search’s key words used were *Magnetic resonance imaging, tomography, computed tomography, CT, cone-beam CT, registration, integration, merging, correlation, fusion, superimposition, image-processing, matching, temporomandibular joint, TMJ, temporomandibular disorder, TMD, craniomandibular disorder, TMJ articular disc, TMJ articular disk.*

MESH keywords and truncated terms were searched with help of a librarian. In addition, manual search of the references in the identified articles was performed to avoid missing relevant articles. Additional file 1 shows the specific combination of the search terminology in different databases.

## Inclusion and exclusion criteria

Studies of different designs (e.g., clinical trials, cohort studies, case–control studies, cross-sectional studies, prospective and retrospective studies, case series/reports) reporting MRI and CT/CBCT image registration for TMJ concerns were included. Reviews, editorials, letters, published errata and historical articles were not included. Articles describing multimodal image registration concerning head and neck oncology were excluded.

## Screening process and data collection

Three independent reviewers (M.A., H.S & N.A.) screened the search data thoroughly and identified the relevant abstracts for full-text article evaluation. When in doubt or unclear from the abstract, the full-text article was selected for evaluation. Preliminary selected abstracts/articles, were reviewed according to the inclusion/exclusion criteria. No clear conflict in the article selection between the two reviewers was reported. Image characteristics and registration type for the included studies were collected and summarized in Table 1.

**Table 1:** Description of the finally included articles

Article	Subjects	Image characteristics	Registration model	Measured outcome
Lin et al. 2008 [22]	1 patient (2 TMJs)	CT: DICOM files. • GE® multilayer spiral CT scanner; 120 kv; 250 mA; slice thickness 0.6 mm. • FOV, matrix size & voxel size were not reported. • Supine scanning position.	• Extrinsic registration model (14 radio-opaque fiducial markers). • Dicom Works® V1.3.5 software.	• Visualize 3D model of TMJ.
		MRI: DCOM files. • Signa® 1.5 T MRI scanner. • T1-weighted image; TR 23 ms; TE 4.6 ms; FOV 25 cm; Matrix 256X128; slice thickness 1.5 mm. • Supine scanning position. • Type of surface coil & voxel size were not reported.		
Dai et al. 2012 [19, 20]	1 patient (one side of TMJ)	Contrast-enhanced CT: DICOM files. • Philips® multilayer spiral CT scanner; 140 kv; 287 mA; slice thickness 1.25 mm; matrix size 512X512. • FOV 23.8 cm; pixle size 0.47 mm. • Contrast agent (Inhexol 300 mg I/ml) Supine scanning position.	• 2D sagittal slices were manually superimposed. • Photoshop® software.	• Matched 2D sagittal slices of MRI and CT of a TMJ to visualize fused image of both modalities.
		MRI: DICOM files. • Signa® 1.5 T MRI scanner. Head surface-coil. • T1-weighted image; TR350-550 ms; TE13-20 ms; Matrix 512X512; slice thickness 4 mm. • Contrast-enhanced T1-weighted image; TR2000-3000 ms; TE15-40 ms; Matrix 512X512; slice thickness 4 mm. (Gadopentetate dimeglumine 0.1mmL/kg). • T2-weighted image; TR 2800-5000 ms; TE 100-120 ms; FOV 24 cm; Matrix 512X512; slice thickness 4 mm. • Supine scanning position.		
Al-Saleh et al. 2015 [15]	10 patients with TMD symptom. (20 TMJs)	CT: DICOM files. • i-CAT® CBCT scanner; 120kv; 5 mA; scan time 9 sec; slice thickness 0.3 mm; matrix size 512X512. • FOV 17X23cm; voxel size 0.3 mm^3^. • Upright scanning position.	• Extrinsic marker-based registration. (5 radio-opaque fiducial markers) • Intrinsic registration (Mutual information-based registration). • Mirada® software.	• Qualitative assessment of the registration models. • Assess the reliability of evaluating TMJ disc position and osseous pathology in 20 TMJs.
		MRI: DCOM files. • Seimens® 1.5 T MRI scanner. Head surface coil. • T1-weighted image; TR 13 ms; TE 4.8 ms; FOV 46X36cm; Matrix 256X128; slice thickness 1 mm; voxel size 1 mm^3^. • Supine scanning position.		

*Abbreviation: TMJ* temporomandibular joint, *CT* computed tomography, *MRI* magnetic resonance imaging, *DICOM* digital imaging and communication in medicine, *FOV* field of view, *TR* repetition time, *TE* echo time, *kv* kilovoltage, *mA* milliAmber

## Results

### Data searched

**Fig. 1:**
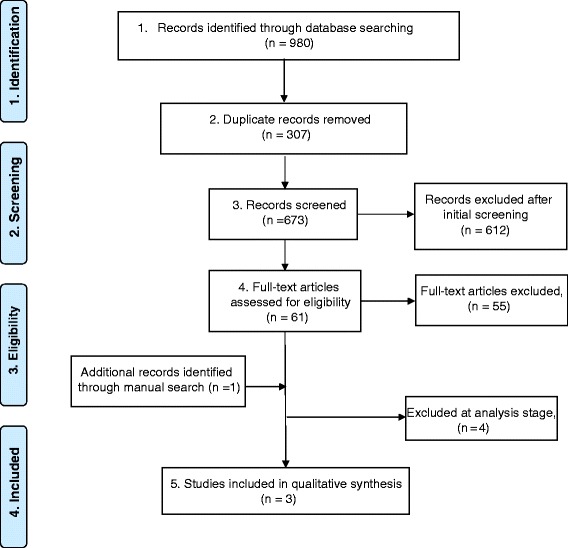
PRISMA 2009 Flow diagram

The database search resulted in a total of 673 articles. The initial review of the titles and abstracts resulted in 61 articles that were considered for full-text review. The full-text review resulted in 6 articles [15, 17–21]. One more article was identified by manual search [22]. Figure 1 demonstrates a flow chart of the articles selection process. Only 3 articles met the inclusion criteria of this review. The 4 remaining articles from the final selection phase were excluded for the following reasons:

1. Measure accuracy of different multimodal image registration techniques [17, 18].
2. Introducing multimodal image registration to visualize the tumors in the head and neck region [20, 21].

### Characteristics of the included articles

All included articles were published within the last 7 years. Two articles described MRI to CT multimodality image registration as a complementary tool to visualize TMJ. Both articles used images of one patient only to introduce the complementary concept of MRI-CT fused image. One article assessed the reliability of using MRI-CBCT registration to evaluate the TMJ disc position and osseous pathology in 20 TMJ’s for 10 temporomandibular disorder (TMD) patients. Table 1 shows the imaging protocols and measured outcomes of the included articles.

### Discussion

#### Multimodal image registration

The essential goal of merging two images from different modalities is to utilize the complementary nature of the displayed information. Proper registration of the different images is crucial especially when used for clinical applications. The process of image registration is composed of two major steps: the first step is the spatial alignment of the target images, which is commonly defined as “registration, and the second is the fused display of the target images, which is defined as “fusion”. Mistakenly, different terminologies have been inter-changeably used in the literature to describe a single step process: such as superimposition, matching, integration, merging and correlation.

**Fig. 2:**
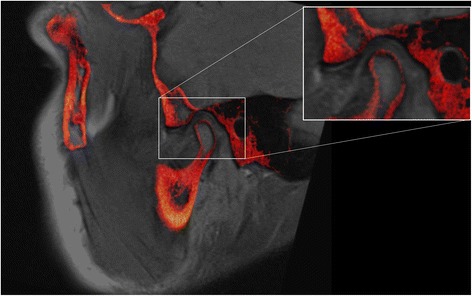
Sagittal view of registered PD-weighted MRI (grey color) and CBCT image (Red color) using maximum mutual information algorithm (intrinsic based registration). The inset shows close-up of the TMJ with excellent superimposition of the TMJ anatomical tissues, despite the different receivers, FOV size, voxel size, voxel value, image-acquired orientation, slice thickness, image resolution and field inhomogeneity

According to van den Elsen et al. and Maintz et al., [23, 24] the registration process was classified into intrinsic and extrinsic models. The intrinsic model depends on anatomical landmarks and segmented bodies or voxel values. The extrinsic model depends on fiducial markers that are either invasively screwed into the tissues or non-invasively attached to the surface skin. Screw-mounted fiducial markers have been considered a gold standard approach for many years to measure the accuracy of the registration process. However, the invasiveness of this approach limits its use to surgical procedures and in-vitro experiments. Anatomical landmarks in the intrinsic registration models are often conspicuous and easy to locate in the human head, however; registration of large tissues in complex regions requires detection of a large number of anatomical landmarks. User interaction is also required to identify the landmarks, which can implicate an operator-bias especially with inexperienced operators. Due to the high degree of similarity between same modality images, monomodal image registration is considered a much easier process than multimodality image registration. In multimodality image registration, such as MRI and CT or CBCT, identifying matched anatomical landmark is a challenging task. Another intrinsic approach is using voxel values (gray values) of the image to spatially align the center of gravity and principal orientation of two images. Using the full image content of gray values in a relative entropy histogram, a method known as “maximization of mutual information”, is a conceptually appealing technique due to its flexibility, easy implementation, automatic and fast use in multimodal image registration (Fig. 2). However, accuracy concerns and sophisticated computational requirements/costs have delayed the clinical application of this registration technique.

For TMJ pathology, MRI or CBCT are the choice of diagnostic imaging depending on availability and the therapeutic indication. Despite the advancement in MR imaging quality, it has not entirely overcome the limitations of the low quality presentation of the complex osseous structure of the TMJ. CBCT is superior at identifying cortical bone contouring, remodeling, developmental abnormality and pathological changes. Both imaging techniques have their limitations and remain complementary to each other in the TMJ diagnostic field.

#### Accuracy of the MRI-CT/CBCT image registration

Registration technique accuracy is a substantial issue when it comes to multimodality image registration. MRI-CT image registration, using maximum mutual information, have been proven accurate in many medical-imaging related studies [25–28]. The linear measurement error (target error) ranged between 0.4-1.6 mm when registered images in the brain, skull and nasopharynx regions. Three studies have reported the accuracy of registration of MRI to CBCT images [17, 18, 29]. Pawiro et al. used fixed fiducial markers, to a cadaver swine head as a gold standard, to measure the accuracy of mutual information based registration of MRI to C-arm CBCT [17]. The registration target error ranged between 0.62 ± 3.19 mm to 1.5 ± 2.3 mm. Tai et al. used a complicated procedure, which involved multiple steps in five different computational software products, to register large FOV 3D MRI to CBCT image [18]. Although this registration technique was cumbersome and somewhat impractical for clinical use, the authors reported a small target error 0.29-0.71 mm when measured against orthodontic dental models. Al-Saleh et al. used fixed fiducial markers to 5 cadaver swine heads to measure the linear target error of MRI-CBCT image registration [29]. The authors’ findings demonstrated a small linear target error (0.2 ± 1.2 mm) when compared to a laser scanner ground truth value. The accuracy of the multi-modality rigid registration has been proven accurate and accessible in the modern advanced imaging technology.

#### Review included articles

Lin et al. was the first to explore the 3D rendering of mandible from MRI and CT registered images [22]. One volunteer was scanned in MRI and CT scanner with 12 fiducial markers attached to the facial skin-surface. The centroids of the markers were identified to detect the center of gravity and spatial relation required for rigid registration. It was not clear how the centroids of the spherical markers were detected, or type of images that were utilized to detect the markers centroid. The authors did not describe the type of the surface coil used for MRI or the voxel size difference between the MRI and CT. Moreover, the registration algorithm/ methods, accuracy, or operator’s bias to manually detect the markers’ centroids were not reported. Extrinsic marker-based registration is rapid and conceptually straightforward, but lacks accuracy. Registration target errors, due to marker displacement (especially when attached to skin), patient position and movement, are not possible to control and substantially affect the registration function. The article’s main objective was to draw the readers’ attention to the feasibility of the MRI-CT registration process and its potential in TMJ anatomical screening. However, the report was simple and lacked details of technical and clinical reporting.

In a brief clinical report, Dai et al. [19] highlighted the importance of merging the MRI and CT images to visualize TMJ tissues. The authors chose one sagittal slice of TMJ MRI and CT images from a previous study, as an example, to illustrate a hybrid image of TMJ via Photoshop® software. Since the image processing applied was not a real registration of two images, the authors indicated in their report that the method was not accurate, and it was merely an example of a future endeavor.

Al-Saleh et al. published the first study that employed MRI and CBCT registered images to assess diagnostic reliability of TMJ pathology [15]. Three radiologists evaluated the quality of two techniques of image registration, extrinsic (fiducial marker-based) versus intrinsic (voxel value mutual information based) in 20 TMJ images. The authors reported poor quality and inaccurate extrinsic MRI-CBCT registration when using 5 skin surface attached markers. The poor alignment of the MRI and CBCT images was attributed to the displacement of the markers, and different patient positioning during imaging. Patients were at supine position during MRI and upright position during CBCT imaging. Matching surface markers seems to be insufficient nor reliable. In contrast, the mutual-information based registration was found to be accurate by all radiologists with high intra- and inter-examiner agreement. Moreover, TMJ osseous pathology and articular disc positon were assessed by all radiologists in 3-interval time. The study found that registered MRI-CBCT images have improved the consistency among radiologists in TMJ disc position evaluation. Although that study did not report the actual registration algorithm or the registration linear target error, it highlighted the importance of viewing well-defined osseous contours and articular disc tissue in one image [15]. Fused MRI and CBCT images have better diagnostic value than the value of each image alone. Several challenges in multimodality image registration starting with, but not limited to, the different receivers, FOV, voxel size, voxel value, image-acquired orientation, slice thickness, image resolution, field inhomogeneity and image artifacts, were largely overcome with the recently introduced robust registration model (mutual information). Although mutual information based image registration is a popular technique in medical image processing, it has not yet been explored in the dental field except for two studies, the one by Al-Saleh et al. [15] and another one for monomodality registration (i.e. two CBCT’s) by Choi and Mah [14]. In addition, the study had a small sample size that could have biased the reported results.

Unlike the medical field, studies about the MRI-CT/CBCT image registration are sparse in the field of dentistry. Out of three studies included in this review, [15, 19, 22] only one study utilized the MRI-CBCT image registration for clinical investigation [15]. The need for well-designed studies in this area is clear.

Multimodality MRI-CBCT image registration has potential to meet clinical needs for simultaneous evaluation of soft and hard tissues at complex structures such as the TMJ, in the field of dentistry and craniofacial surgery. However, multimodal image registration technology is relatively young and there is little evidence regarding its clinical use in many areas in dentistry. Challenges, such as complexity and accuracy concerns for the different registration techniques including different imaging protocols have been improved over the past few years, but have not yet led to general clinical applicability. This review highlights the need for further work in the field of dental multimodality image fusion.

## Future recommendations

To explore the accuracy and clinical application of MRI-CBCT image registration in the field of craniofacial and TMJ. This review suggests the following:

1. Measure the accuracy of the MRI-CBCT mutual information algorithm using a gold standard tool independent of MRI or CBCT.
2. Test the usefulness of the fused MRI-CBCT in evaluating the TMJ among practitioners with different levels of expertise.
3. Explore objective tools to measure disc position or changes in relation to osseous structure using 3D volume rendering.

## Conclusions

There are very limited studies of MRI-CT/CBCT registration, with data insufficient to reach a conclusion regarding its accuracy or clinical use in the temporomandibular joints.

Mutual information based registration seems a promising technique, and exploring its accuracy and applications for TMJ analysis would be worthwhile in larger studies.

## Additional file

Additional file 1: Search strategy. (DOCX 22 kb)
