# Supplementary material for: MRI and CBCT image registration of temporomandibular joint: a systematic review
Source: J Otolaryngol Head Neck Surg. 2016 May 10;45:30. doi: 10.1186/s40463-016-0144-4 (PMC4863319; doi:10.1186/s40463-016-0144-4)
Supplement: Additional file 1: — Search strategy. (DOCX 22 kb) [file 40463_2016_144_MOESM1_ESM.docx]

**Appendix: Search Strategy**

| **Database** | **Keywords** | **Results**  **After abstract screening** | **Selected** |  |
| --- | --- | --- | --- | --- |
| Ovid MEDLINE(R) In-Process & Other Non-Indexed Citations, Ovid MEDLINE(R) Daily and Ovid MEDLINE(R) <1946 to Present> | 1     exp Magnetic Resonance Imaging/ or MRI.mp. (396001) 2     exp Tomography, X-Ray Computed/ or CT.mp. (439315) 3     exp Cone-Beam Computed Tomography/ or CBCT.mp. (5907) 4     cone beam CT.mp. (2084) 5     1 or 2 or 3 or 4 (753812) 6     TMJ.mp. or exp Temporomandibular Joint/ (14080) 7     exp Temporomandibular Joint Disorders/ or TMD.mp. or exp Temporomandibular Joint Dysfunction Syndrome/ (16654) 8     Craniomandibular [disorder.mp](http://disorder.mp/). or exp Craniomandibular Disorders/ (15165) 9     internal [derangement.mp](http://derangement.mp/). (1088) 10     Temporomandibular Joint Disc.mp. or exp Temporomandibular Joint Disc/ (1638) 11     temporomandibular Joint Disk.mp. (110) 12     6 or 7 or 8 or 9 or 10 or 11 (24634) 13     exp Image Processing, Computer-Assisted/ or [registration.mp](http://registration.mp/). (237947) 14     Integration.mp. (124984) 15     [merging.mp](http://merging.mp/). (5001) 16     [fusion.mp](http://fusion.mp/). (239841) 17     [matching.mp](http://matching.mp/). (54912) 18     [superimposition.mp](http://superimposition.mp/). (2655) 19     13 or 14 or 15 or 16 or 17 or 18 (649700) 20     5 and 12 and 19 (464) | 11 | 4 |  |
| Embase  <1974 to 2016 January 18> | 1     exp Magnetic Resonance Imaging/ or MRI.mp. (648560) 2     exp Tomography, X-Ray Computed/ or CT.mp. (811160) 3     exp Cone-Beam Computed Tomography/ or CBCT.mp. (8822) 4     cone beam CT.mp. (3525) 5     1 or 2 or 3 or 4 (1274044) 6     TMJ.mp. or exp Temporomandibular Joint/ (15601) 7     exp Temporomandibular Joint Disorders/ or TMD.mp. or exp Temporomandibular Joint Dysfunction Syndrome/ (13781) 8     Craniomandibular [disorder.mp](http://disorder.mp/). or exp Craniomandibular Disorders/ (11521) 9     internal [derangement.mp](http://derangement.mp/). (1212) 10     Temporomandibular Joint Disc.mp. or exp Temporomandibular Joint Disc/ (12017) 11     temporomandibular Joint Disk.mp. (107) 12     6 or 7 or 8 or 9 or 10 or 11 (24743) 13     exp Image Processing, Computer-Assisted/ or [registration.mp](http://registration.mp/). (123359) 14     [merging.mp](http://merging.mp/). (5516) 15     [fusion.mp](http://fusion.mp/). (197471) 16     [matching.mp](http://matching.mp/). (73230) 17     [superimposition.mp](http://superimposition.mp/). (3017) 18     Integration.mp. (140940) 19     13 or 14 or 15 or 16 or 17 or 18 (532017) 20     5 and 12 and 19 (214) | 4 | 1 |  |
| EBM Reviews - Cochrane Database of Systematic Reviews <2005 to January 13, 2016>, EBM Reviews - ACP Journal Club <1991 to December 2015>, EBM Reviews - Database of Abstracts of Reviews of Effects <2nd Quarter 2015>, EBM Reviews - Cochrane Central Register of Controlled Trials <December 2015>, EBM Reviews - Cochrane Methodology Register <3rd Quarter 2012>, EBM Reviews - Health Technology Assessment <4th Quarter 2015>, EBM Reviews - NHS Economic Evaluation Database <2nd Quarter 2015> | 1     (Magnetic Resonance Imaging or MRI).mp. [mp=ti, ot, ab, tx, kw, ct, sh, hw] (11865) 2     (CT or computed tomography).mp. [mp=ti, ot, ab, tx, kw, ct, sh, hw] (61531) 3     (CBCT or Cone Beam Computed Tomography).mp. [mp=ti, ot, ab, tx, kw, ct, sh, hw] (165) 4     (TMJ or Temporomandibular Joint).mp. [mp=ti, ot, ab, tx, kw, ct, sh, hw] (861) 5     (Temporomandibular Joint Disorder or TMD).mp. [mp=ti, ot, ab, tx, kw, ct, sh, hw] (342) 6     Temporomandibular Joint Disc.mp. [mp=ti, ot, ab, tx, kw, ct, sh, hw] (72) 7     Temporomandibular Joint Disk.mp. [mp=ti, ot, ab, tx, kw, ct, sh, hw] (12) 8     Craniomandibular [disorder.mp](http://disorder.mp/). [mp=ti, ot, ab, tx, kw, ct, sh, hw] (10) 9     Image [processing.mp](http://processing.mp/). [mp=ti, ot, ab, tx, kw, ct, sh, hw] (1751) 10     Registration.mp. [mp=ti, ot, ab, tx, kw, ct, sh, hw] (14090) 11     Integration.mp. [mp=ti, ot, ab, tx, kw, ct, sh, hw] (2271) 12     Merging.mp. [mp=ti, ot, ab, tx, kw, ct, sh, hw] (92) 13     Fusion.mp. [mp=ti, ot, ab, tx, kw, ct, sh, hw] (3891) 14     Matching.mp. [mp=ti, ot, ab, tx, kw, ct, sh, hw] (5429) 15     Superimposition.mp. [mp=ti, ot, ab, tx, kw, ct, sh, hw] (53) 16     1 or 2 or 3 (71109) 17     4 or 5 or 6 or 7 or 8 (982) 18     9 or 10 or 11 or 12 or 13 or 14 or 15 (26994) 19     16 and 17 and 18 (4) | 0 | 0 |  |
| Scopus   1. January 18 | 1. History Search TermsTITLE-ABS-KEY ( "Cone beam CT" OR cbct ) 6,364 document results 2. History Search Terms( TITLE-ABS-KEY ( "Magnetic Resonance Imaging" OR mri ) ) OR ( TITLE-ABS-KEY ( "Computed Tomography" OR ct ) ) OR ( TITLE-ABS-KEY ( "Cone beam CT" OR cbct ) ) 998,049 document results 3. History Search TermsTITLE-ABS-KEY ( "Temporomandibular Joint" OR tmj OR "Temporomandibular Joint Disorder" OR tmd OR "Craniomandibular Disorder" OR "Temporomandibular Joint Disc" OR "Temporomandibular Joint Disk" ) 30,918 document results 4. History Search TermsTITLE-ABS-KEY ( registration OR "image processing" OR superimposition OR fusion OR matching OR integration OR merging ) 1,704,426 document results 5. History Search Terms( ( TITLE-ABS-KEY ( "Magnetic Resonance Imaging" OR mri ) ) OR ( TITLE-ABS-KEY ( "Computed Tomography" OR ct ) ) OR ( TITLE-ABS-KEY ( "Cone beam CT" OR cbct ) ) ) AND ( TITLE-ABS-KEY ( "Temporomandibular Joint" OR tmj OR "Temporomandibular Joint Disorder" OR tmd OR "Craniomandibular Disorder" OR "Temporomandibular Joint Disc" OR "Temporomandibular Joint Disk" ) ) AND ( TITLE-ABS-KEY ( registration OR "image processing" OR superimposition OR fusion OR matching OR integration OR merging ) ) **298** document results. | 1 | 1 |  |
| Subtotal |  |  | 6 |  |
| Repeated |  |  | 3 |  |
| Total to be analyze for inclusion |  | 16 | 3 |  |
| Manual search to be analyze for inclusion |  | 00 | 00 |  |
| Total |  |  | 3 |  |
